# The p53/miR-17/Smurf1 pathway mediates skeletal deformities in an age-related model via inhibiting the function of mesenchymal stem cells

**DOI:** 10.18632/aging.100728

**Published:** 2015-03-07

**Authors:** Wenjia Liu, Meng Qi, Anna Konermann, Liqiang Zhang, Fang Jin, Yan Jin

**Affiliations:** ^1^ State Key Laboratory of Military Stomatology, Center for Tissue Engineering, School of Stomatology, The Fourth Military Medical University, Xi'an, Shaanxi 710032, People's Republic of China; ^2^ Research and Development Center for Tissue Engineering, Fourth Military Medical University, Xi'an, Shaanxi 710032, People's Republic of China; ^3^ Department of Orthodontics, Medical Faculty, University of Bonn, Bonn, Germany; ^4^ State Key Laboratory of Military Stomatology, Department of Orthodontic, School of Stomatology, The Fourth Military Medical University, Xi'an, Shaanxi 710032, People's Republic of China

**Keywords:** Aging, mesenchymal stem cells, osteogenesis, p53, miR-17

## Abstract

Osteoporosis is an age-related progressive bone disease. Trp53 (p53) is not only a famous senescence marker but also a transcription regulator which played a critical role in osteogenesis. However, how p53 contributes to the bone mass loss in age-related osteoporosis is still unclear. Here, we found that bone mass and osteogenic differentiation capacity of mesenchymal stem cells (MSCs) is significantly reduced with advancing age. Serum levels of TNF-α and INF-γ and senescence-associated ß-galactosidase, p16, p21 and p53 are significantly increased in elder mice, but antipodally, osteogenic marker expression of Runx2, ALP and osterix are reduced. Overexpression p53 by lentivirus inhibits osteogenesis in young MSCs in culture and upon implantation in NOD/SCID mice through inhibiting the transcription of miR-17-92 cluster, which is decreased in old mice. In addition, miR-17 mimics could partially rescue the osteogenesis of old MSCs both *in vitro* an *in vivo*. More importantly, Smurf1 as a direct target gene of miR-17, plays an important role in the p53/miR-17 cascade acting on osteogenesis. Our findings reveal that p53 inhibits osteogenesis via affecting the function of MSCs through miRNA signaling pathways and provide a new potential target for treatment in future.

## INTRODUCTION

Age-related diseases include cancer, cardiovascular disease, diabetes, various neurodegenerative diseases [1], and especially osteoporosis. A reduction in these age-related diseases will not only enhance the quality of life but also reduce the overall burden to society and families. Bone marrow mesenchymal stem cells (BMMSCs) are pluripotent cells with the potential for self-renewal and multiple differentiations into other cell types, dedicating them for regenerative medicine and tissue engineering, as they provide tissue maintenance and repair after damaging insults [2, 3]. In addition, bone homeostasis is supposed to fundamentally depend on the transformation potential of BMMSCs, particularly in this case different into osteoblastic cells [4]. However, the qualification of BMMSCs for recovery of multiple tissue systems is contingently compromised with age[3, 5]. As senescent cells can remain and agglomerate in tissues in contrast to apoptotic cells that are immediately removed by host-defensively processes, their existence can profoundly affect homeostatic mechanisms of the whole body [6-8].

The age-related restrictions of BMMSCs take special effect on their osteogenic and adipogenic potential and imply expression changes of associated marker genes and of senescence-related molecules [2, 9, 10]. Osteogenesis and adipogenesis appear to decline with age and passage of the cultivated cells [11-14], and the loss of osteogenic potential is presumably connected with an inhibited upregulation of key osteogenic transcription factors due to an altered p53 level [4, 15], which commonly recognized as tumor suppressing gene, is also one of the most widely studied genes in aging. A mutant p53 heterozygote mouse model developed by Donehower et al. (p53^+/m^ mice) demonstrated increased osteoporosis, organ atrophy, diminished stress tolerance and shortened life span in the p53^+/m^ mice as compared to wild type littermates [16]. In addition, researches revealed that p53 occupies a dual role in its mode of operation, as it manifests both sequence-specific DNA-binding mechanisms and transcription-independent capacities and can operate as transcriptional activator as well as repressor on certain genes [17-19].

MiRNAs consisting of 21-23 nucleotide RNA molecules not only provide stability and translational efficiency of target mRNAs, but also involved in cellular differentiation, proliferation and apoptosis [20-22]. Stem cell lineage commitment into osteoblasts is likewise governed by diverse miRNAs, either in terms of inhibition or enhancement of osteogenesis, by miR-204/211 suppressing Runx2 or by miR-20a activating BMP signaling for example [23, 24]. Our previous research revealed that miR-17 acted as a negative regulator of osteogenesis in a physiological microenvironment, but contrarily as promoter for osteoblastic commitment of tissue-specific MSCs under inflammatory pathological conditions due to targeting different genes [25, 26]. Recent investigations show that p53 can repress miR-17-92, a cluster of 7 microRNA (miRNAs), on transcriptional level via action on p53-binding site located on the proximal part of the miR-17-92 promoter region [27, 28]. However, the detailed mechisnam of how p53 contributes to bone mass loss in age-related osteoporosis is still unclear. In this study, we choose natural aging mice as our study model and demonstrate that BMMSCs from 16-month old (old) mice express decreased osteogenic differentiation capacity and increased senescence markers, expecially p53. Overexpression p53 in 4-month-old (young) BMMSCs could inhibit the osteogensis of them both *in vitro* and *in vivo*. In addition, miR-17 mimics particular rescue the osteogenesis of old BMMSCs both *in vitro* and *in vivo*. We also elucidated the underlying mechanism that p53 restained the osteogenesis via modulating the trancription of pri-miR-17 and then affecting the expression of Smurf1, a direct target of miR-17. Our study aims to reveal a novel mechanism of age-related osteoporosis and provide a new potential target for treatment in future.

## RESULTS

### Bone formation and osteogenic differentiation capacity are significantly reduced with advancing age

**Figure 1:**
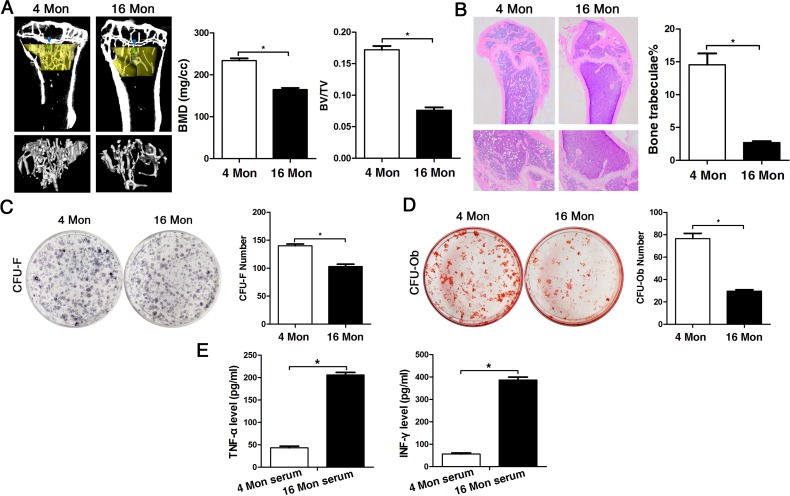
The osteogenic capacity of old mice is significantly reduced both *in vivo* and *in vitro*. Statistically analyzed values show the mean ± SD (n=10). * *p* < 0.05. **(A)** Micro-CT analysis of trabecular bone mass in the tibiae of 4 (young) and 16 month-old (old) mice. Quantitative analyses were performed via volumetric bone mineral density (BMD) and trabecular bone volume fraction (BV/TV) measurements. **(B)** HE stainings of histological sections from femur derived from young and old mice for detection of the number of bone trabeculae. **(C-D)** Representative images of the CFU-F assay for determination of proliferation capacity and of the CFU-Ob assay for osteogenic differentiation ability of BMMSCs obtained from young and old mice and stained with crystal violet and alizarin red, respectively. CFU efficiency was determined by the number of colonies relative to the total number of seeded cells in each plate. **(E)** Serum levels of TNF-α and INF-γ in young and old mice determined via ELISA. Results are expressed as pg/ml.

In order to assess bone formation ability with advancing age *in vivo*, bone mass was analyzed in 4 (young) and 16-month-old (old) mice via micro-CT analysis as much as via histological staining of femur tissue sections. Both methods and the corresponding analyses comprising BMD and BV/TV measurements as much as determination of the number of bone trabeculae revealed that bone mineral density was decreased in old mice relative to young mice with significant loss in trabecular bone volume and in the number of trabeculae (Fig. 1A-B). In BMMSCs derived from young and old mice, the CFU assay showed that the number of CFU-F and CFU-Ob of old mice was significantly less compared to young mice (Fig. 1C-D), especially the CFU-Ob number, thus confirming the *in vivo* data. In addition, analyses on serum levels of TNF-α and IFN-γ both in young and old mice revealed significantly higher values of these cytokines in elder mice (Fig. 1E), suggesting more inflammation with advancing age.

**Figure 2:**
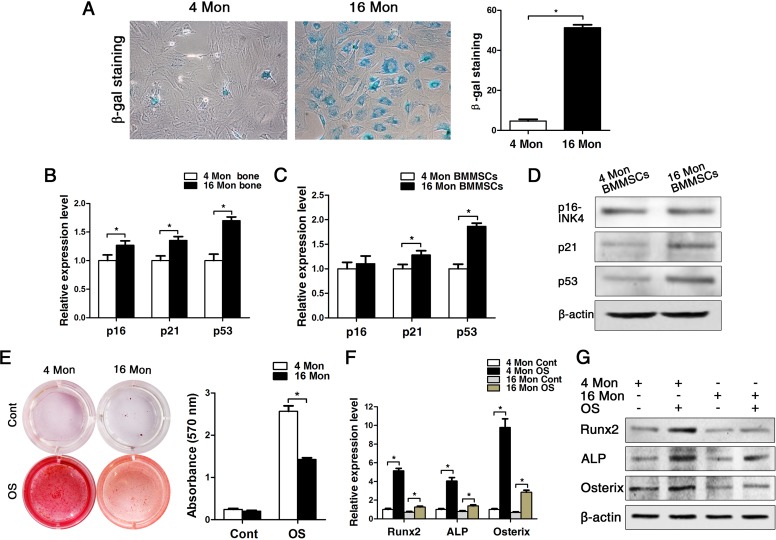
BMMSCs from old mice express higher levels of senescence markers and lower osteoblast markers compared to young ones. Statistically analyzed values show the mean ± SD (n=10). * *p* < 0.05. **(A)** In vitro staining of the senescence-related marker ß-galactosidase in BMMSCs cultures derived from young and old mice. Quantitative analysis of the total number of positively stained cells. **(B-C)** Real-time PCR analyses on whole bone tissue extracts (**B**) and on BMMSCs (**C**) for the senescence-related genes p16, p21 and p53. Normalization to ß-actin. **(D)** The western blot showed that the protein level changed as the mRNA. **(E)** Alizarin red staining of BMMSCs from young and old mice osteogenically induced for 14 d. Cont = Control, OS = osteogenically induced. **(F-G)** Real-time PCR and western blot analyses on BMMSCs for the osteogenic markers Runx2, ALP, osterix. Normalization to ß-actin.

In old mice, senescence-associatedß-galactosidase was detectable to a significantly greater amount compared to cells from young mice, and the senescence-associated markers p16, p21 and p53 were also significantly increased both in bone tissues and in BMMSCs on gene protein level (Fig. 2A-D). Additionally, the osteogenic differentiation capacity of BMMSCs derived from old mice was significantly reduced compared to the ones obtained from young mice, which was evident from the results of the alizarin red staining as much as the gene and protein expression analyses of osteogenic marker expression, namely Runx2, ALP and osterix (Fig. 2E-G). In addition, the proliferation capacity of BMMSCs from old mice was also lower than BMMSCs from young mice, as indicated by MTT and flow cytometric cell cycle analysis (Supplementary Fig. 1).

### p53 is causative for inhibited osteogenesis in BMMSCs

**Figure 3:**
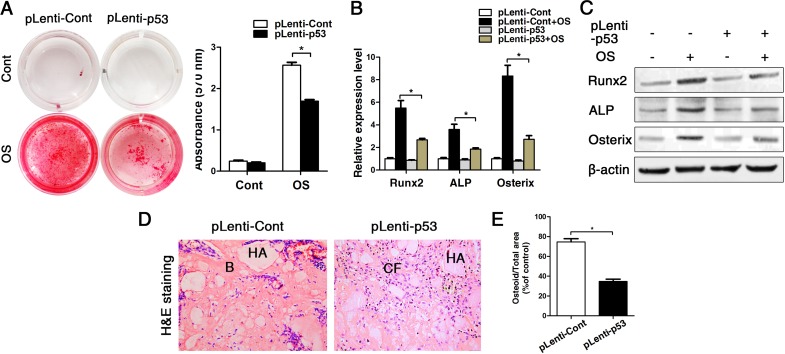
Overexpression of p53 changed the phenotype of young BMMSCs into old BMMSCs. BMMSCs from young mice were lentivirally transduced to upregulate the expression level of p53 (= pLenti-p53) or were transduced as lentiviral control (= pLenti-Cont). Statistically analyzed values show the mean ± SD (n=10). * *p* < 0.05. **(A)** Alizarin red staining of pLenti-p53 and of pLenti-Cont after osteogenic inducing for 14 days. Cont = Control, OS = osteogenically induced. The values show the mean ± SD (n=10). * *p* < 0.05. (**B-C)** Real-time PCR and western blot analyses on BMMSCs with lentiviral transduction (pLenti-p53 and pLenti-Cont) and with/without osteogenic induction for the osteogenic markers Runx2, ALP, osterix. Normalization to ß-actin. (**D-E)** Histological analyses and corresponding statistical analysis of tissue sections from subcutaneous pockets on the backs of 6-week-old NOD/SCID mice with implanted HA/TCP ceramic particles mixed with BMMSCs from young mice with lentiviral transduction of p53 and control.

As we observed that old BMMSCs express significantly higher gene and protein levels of the senescence-related markers p21, p16 and most substantially p53, we further investigated the relationship between p53 and osteogenic differentiation capacity of BMMSCs. We constructed the lentiviral vector to induce a stable up-regulation of p53 in BMMSCs. The lentiviral construct (pLenti-p53) increased the p53 expression level more than 5-fold compared with the control (Supplementary Fig. 2). Then wecultured the cells in osteogenic differentiation medium for an additional 14 days. Alizarin red staining and the expression of ALP, Runx2 and osterix showed that the osteogenic differentiation of BMMSCs from young mice was suppressed after transducing pLenti-p53 *in vitro* (Fig. 3A-C). Then, we expended this study for ectopic bone formation *in vivo*. The BMMSCs transduced with pLenti-p53 or control vector were loaded onto HA/TCP powder scaffolds and implanted in NOD/SCID mice for 8 weeks. The control group was found to form a considerable amount of bone tissue around the HA/TCP powder. The new bone tissue was stained red using HE staining. The pLenti-p53-transduced BMMSCs only formed some threadlike collagen fibers around the surface of the HA/TCP powders. Osteoid formation was decreased 45% in the implants treated with the pLenti-p53 compared with those treated with the vector control (Fig. 3D-E).

### p53 regulates the osteogenesis of BMMSCs through inhibiting the transcription of miR-17-92 cluster

**Figure 4:**
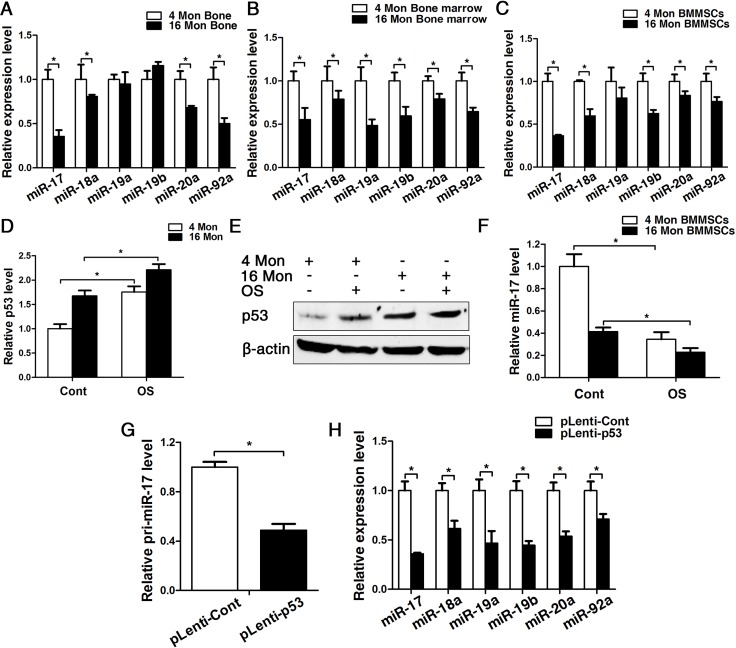
p53 contribute to impaired osteogenesis of BMMSCs via inhibiting the transcription of miR-17-92 cluster. BMMSCs were lentivirally transduced to upregulate the expression level of p53 (= pLenti-p53) or were transduced as lentiviral control (= pLenti-Cont). Statistically analyzed values show the mean ± SD (n=10). * *p* < 0.05. **(A-C)** Real-time PCR analyses for the expression of miR-17, miR-18a, miR-19a, miR-19b, miR-20a and miR-92a in bone (**A**), bone marrow (**B**) and BMMSCs (**C**) of young and old mice. Normalization to ß-actin. **(D-F)** Real-time PCR and western blot analysis of p53 (**D**, **E**) and real-time PCR of miR-17 (F) expression in BMMSCs derived from young and old mice after osteogenic differentiation for 7 d. Normalization to ß-actin and U6. **(G)** Pri-miR-17 transcript analysis by Taqman-based qPCR. Normalization to GAPDH. **(H)** Real-time PCR analysis of the mature miR-17-92 cluster after upregulating P53 for 48 h. Normalization to U6.

Considering that senescence can be correlated to a chronic inflammatory microenvironment (Fig.1E) and as our previous research revealed that miR-17 acts as positive regulator of osteogenesis in an inflammatory microenvironment [25], we investigated the expression pattern of miR-17-92 cluster in BMMSCs from young and old mice. Real-time PCR analyses showed a significant decrease of miR-17, miR-18a, miR-20a and miR-92a in bone tissues, reduction of all family members in bone marrow and reduced expression of miR-17, miR-18a, miR-19a, miR-20a and miR-92a could be observed in BMMSCs (Fig. 4A-C). Importantly, the expression pattern of p53 and miR-17 were exactly opposite in BMMSCs from both 4 and 16-month-old mice during osteogenic differentiation (Fig. 4D-F). Furthermore, the expression of pri-miR-17 and mature miR-17-92 family members significantly decreased upon overexpression of p53, suggesting that p53 can potently block the transcription of miR-17-92 (Fig. 4G, H).

**Figure 5:**
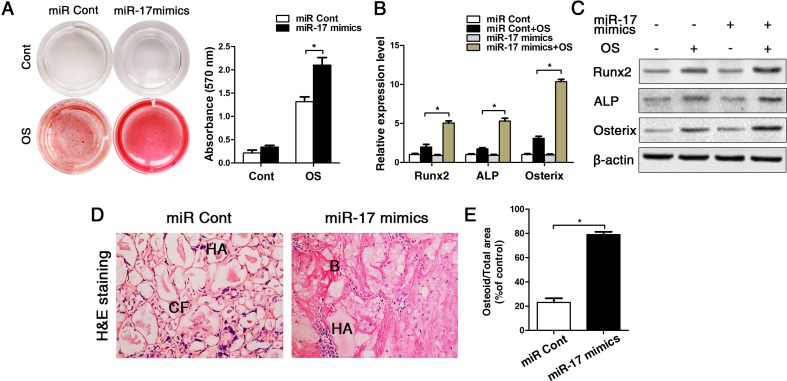
Up-regulation of miR-17 by miR-17 mimics reversed the effect of p53 on inhibiting osteogenic differentiation in old BMMSCs. miR-17 was stable upregulated in BMMSCs by miR-17 mimics (= miR-17 mimics). miRNA control (= miR Cont). Statistically analyzed values show the mean ± SD (n=10). * *p* < 0.05. **(A)** Osteogenic induction for 14 d of BMMSCs derived from old mice and subsequent alizarin red staining resulted in a heightened osteogenic differentiation of BMMSCs with previously upregulated miR-17 expression. **(B-C)** Real-time PCR and western blot analyses on old BMMSCs with miR-17 mimic treatment or miRNA control and with/without osteogenic induction for the osteogenic markers Runx2, ALP, osterix. Normalization to ß-actin. **(D-E)** Histological analysis (D) and corresponding statistical analysis (E) on osteoid formation of tissue sections from subcutaneous pockets on the backs of 6-week-old NOD/SCID mice with implanted HA/TCP ceramic particles mixed with BMMSCs from 16-month-old mice with/without miR-17 upregulation.

Since miR-17 were decreased obvious in old mice (bone, bone marrow and BMMSCs), we next used miR-17 mimics to up-regulated expression of miR-17 in old BMMSCs. Our data showed that the transfection efficiencies of miR-17 mimics and inhibitor persisted at least for 14 d (Supplementary Fig. 3). After osteogenic induction of old BMMSCs for 14 d *in vitro*, alizarin red staining suggested that the osteogenic differentiation of BMMSCs was obviously enhanced after upregulating miR-17 expression (Fig. 5A). These data of the staining results were supported by the transcriptional and protein analyses of the osteoblast-related genes Runx2, ALP and Osterix, which is illustrated in Fig. 5B-C. The *in vitro* results could be substantiated *in vivo*by the HA/TCP transplantation experiments earlier described, as after a transplantation perio d for 8 weeks, BMMSCs from old mice formed plenty of new bony structures around the HA/TCP granules when miR-17 was upregulated in the transplanted cells (Fig. 5D-E).

### Smurf1 plays an important role in the p53/miR-17 cascade acting on osteogenesis of BMMSCs

**Figure 6:**
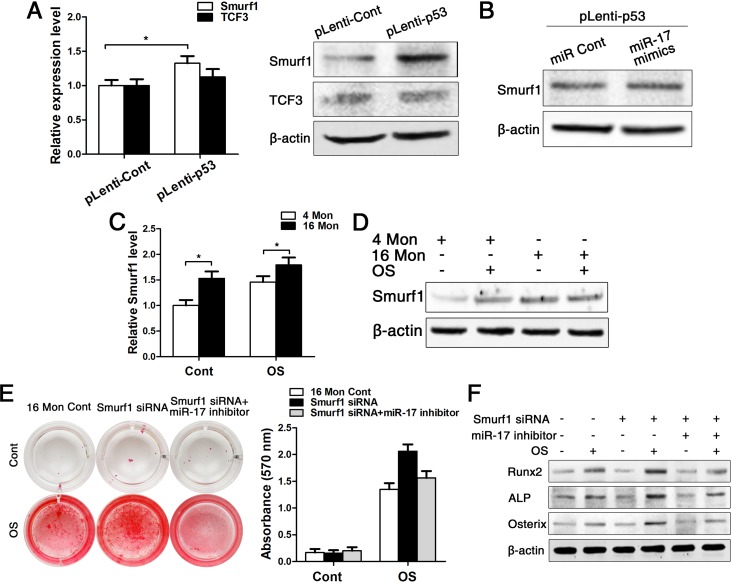
Smurf1 plays an important role in miR-17-mediated osteogenic differentiation of BMMSCs. BMMSCs were lentivirally transduced to upregulate the expression level of P53 (= pLenti-P53) or were transduced as lentiviral control (= pLenti-Cont). miR-17 was stable upregulated in BMMSCs by miR-17 mimics (= miR-17 mimics). miRNA control (= miR Cont). 16 Mon Cont = control BMMSCs, Smurf1 siRNA = downregulated Smurf1 level via si-RNA, miR-17 inhibitor = transfection with anti-miR-17. Statistically analyzed values show the mean ± SD (n=10). * *p* < 0.05. **(A)** Real-time PCR and western blot analysis on the expression of Smurf1 and TCF3 after upregulation of p53 (pLenti-p53) in BMMSCs derived from young mice. Normalization to ß-actin. **(B)** Western blot analysis on the expression of Smurf1. Transfection of miR-17 mimics in stable upregulated p53 BMMSCs derived from young mice. Normalization to ß-actin. **(C-D)** Real-time PCR and western blot analysis of Smurf1 expression in osteogenically differentiated BMMSCs from young and old mice. Normalization to ß-actin. **(E)** Alizarin red staining after osteogenic induction for 14 d of BMMSCs derived from old mice with/without siRNA-downregulated Smurf1 level and with/without transfection with miR-17 inhibitor. **(F)** Western blot analysis on old BMMSCs with/without siRNA-down-regulated Smurf1 level and with/without transfection with anti-miR-17 for the osteogenic markers Runx2, ALP, osterix. Normalization to ß-actin.

**Figure 7:**
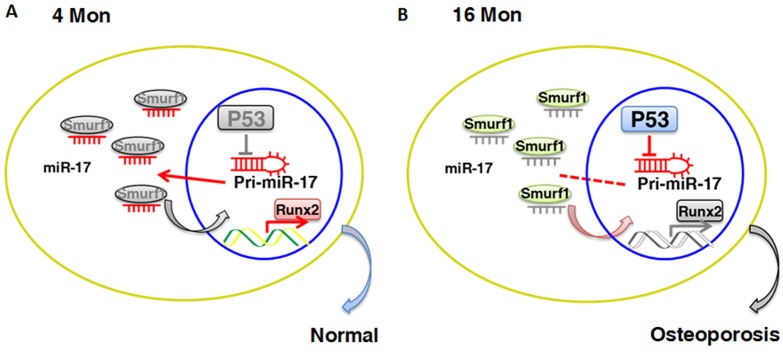
Schematic diagram of p53/miR-17/Smurf1 cascade. **(A, B)** p53 regulates the osteogenic differentiation of BMMSCs through inhibiting transcription of miR-17-92 cluster and subsequent modulating Smurf1, a direct target gene of miR-17, aslo acts as a negative regulator for osteogenic differentiation of mesenchymal stem cells.

As microRNA has to bind to its target gene for regulating cellular characteristics, we subsequently tested the expression of two direct target genes of miR-17, namely TCF3 and Smurf1. Both real-time PCR and western blot data showed that the expression of Smurf1 was increased after upregulating p53, and here especially on protein level, as miRNA acts on post-transcriptional level and mostly affects protein expression. However, the other target gene TCF3 was not changed after upregulating p53 (Fig. 6A). Next, we transfected cells which were stably upregulated p53 with miR-17 mimics for following investigation of Smurf1 expression. The western blot data showed that the Smurf1 level was almost unchanged (Fig. 6B), suggesting that p53 affects the expression level of Smurf1 mainly through miR-17, which is illustrated as an overview model in Fig. 7.

Then, we examined the expression pattern of Smurf1 during osteogenic differentiation of young and old BMMSCs and could surprisingly found that the expression of Smurf1 was increased in both groups upon osteogenic differentiation, although Smurf1 is announced as negative regulator of osteogenesis (Fig. 6C-D). In both the control and the osteogenically induced group, Smurf1 expression was higher in old compared to young BMMSCs. Therefore, we used siRNA to down-regulate the level of Smurf1 in BMMSCs from old mice in a next step (Supplementary Fig. 4). After a 14 d period of osteogenic induction, the alizarin red staining displayed that osteogenic differentiation of old BMMSCs could partially be rescued after downregulating Smurf1 expression. However, this effect disappeared after decrease of miR-17 expression by miR-17 inhibitor, which could be substantiated by gene expression analysis of osteoblast-related genes (Fig. 6E-F).

## DISCUSSION

Senescence of cells and especially of stem cells is an ongoing matter of debate, and its potential impact on tissue homeostasis is up to date still largely obscure. Aggravating this concern is the fact that there is no clear evidence whether the age-related cellular changes observed *in vitro* such as growth stagnation and diminished differentiation capacity also impact tissue homeostasis *in vivo* [3] Additionally, the molecular mechanisms involved in and activated by occurrence of senescence are widely unknown, thus making a disclosure of possible consequences for cellular signaling pathways and the corres ponding effects in tissues almost impossible. Therefore, our study was meant to investigate the cellular characteristics of aging BMMSCs and the resulting changes in their osteogenic potential both *in vivo* and *in vitro*.

Our analyses revealed that *in vivo* bone formation in terms of volumetric bone mineral density and trabecular bone volume fraction in femur tissue sections and *in vitro* correspondingly osteogenic differentiation of murine BMMSCs are reduced with advancing age. BMMSCs from elder mice exhibit an increase in senescence-associated ß-galactosidase, p16, p21, p53 and concurrently a decrease in proliferation and occurrence of osteogenic markers alizarin red, Runx2, ALP and osterix. Interestingly, inflammatory molecules TNF-α and IFN-γ both revealed significantly higher values of these cytokines in elder mice, suggesting more inherent inflammation with advancing age. Previous studies detected a dependence between heightened levels of inflammatory molecules and impaired bone formation capacity in inflammatory bone diseases, and due to our results evidence suggests an intrinsic predisposition for inflammatory priming in senescent cells [29, 30]. In consideration that inflammatory cytokines are qualified to induce inflammatory settings by modulation of miRNAs, our findings are concordant with these results [31, 32]. We could show that p53 inhibits osteogenic differentiation of BMMSCs *in vivo* and that these effects impair osteoid formation about 45% *in vitro* when p53-transfected BMMSCs are loaded on implanted HA/TCP.

Analyses on the molecular pathways involved in this repressive influence of p53 uncovered a reversion of the inhibitory effects of p53 on osteogenesis upon miR-17 overexpression with rescue of osteogenic differentiation of old BMMSCs and vice versa a restraint of miR-17 by p53 overexpression. MiR-17-92 cluster plays an important role in senescence and has a close relationship with p53, as p53 can bind to the promoter region of miR-17-92 for repression of its function, which plays a key role in hypoxia-induced apoptosis [20]. MiR-17 is closely related to TCF3 and Smurf1, two direct target genes in the cells that miR-17 can bind and thus block these negative regulators of osteogenesis [25, 26]. In both our control and osteogenically induced group, Smurf1 expression was higher in old compared to young BMMSCs, and upregulation of p53 increased Smurf1 expression as well. Rising of both p53 and miR-17 at the same time did not alter the level of Smurf1, demonstrate-ing that p53 regulates Smurf1 indirectly by inhibition of miR-17. Contrarily, TCF3 remained unaffected by any of the treatments, providing an indication that the target gene miR-17 binds to is dependent on the microenvironment and the cellular components involved. On the basis of our previous results, miR-17 tends to target TCF3 in the normal microenvironment, while it aims to target Smurf1 under inflammatory conditions [25, 26]. As senescent cells have been shown to display elevated expression profiles of inflammatory molecules, the trend that miR-17 has an affinity for binding Smurf1 can be interpreted in this manner.

In summary, our results illustrate that cellular senescence involves inhibiting effects on osteogenesis *in vitro* as well as *in vivo* with major impact of regulatory mechanisms engaging in miRNA signaling pathways. This knowledge provides the basis for the development of new treatment strategies in age-related changes of bone homeostasis both under physiological and pathological conditions.

## METHODS

### Mice

Twenty 4-month and 16-month old C57/BL6 mice, female, respectively (n=10 specimens per group for histology, n=10 for cell culture) and 6-week old immunocompromised nude mice (CAnN.Cg-Foxn1nu/CrlVr) (n=10 specimens per group), female, were purchased from Vital River Laboratory Animal Technology Co. Ltd. (Beijing, China). All procedures involving animals were approved by the Animal use and Care Committee of the Fourth Military Medical University (license number: SCXK 2007-007).

### Micro-CT analysis

The mice (n=5) from each experimental group were scanned with the Inveon micro-CT system (Siemens Healthcare Diagnostics GmbH, Eschborn, Germany). Cross-sectional volumetric BMD was measured at right tibia mid-diaphysis. Using two-dimensional images, a region of interest in secondary spongiosa was manually drawn near the endocortical surface, and cancellous bone morphometric parameters including BV/TV in % were assessed. Experiments were performed in triplicate.

### Bone histological analysis

The femurs derived from the mice of each experimental group were fixed with 4% paraformaldehyde, decalcified with 10% EDTA (pH 7.0) and embedded in paraffin. For histological analysis, tissue sections were deparaffinized and stained with HE followed by trabecular percentage calculation using Image J software. Experiments were performed in triplicate.

### Isolation of murine BMMSCs

Bone marrow cells (3×10^7^) were flushed out from long bones of each experimental group of mice (n=5) with 2% FBS in PBS. A single-cell suspension of all nucleated cells was obtained by passing bone marrow cells through a 70 μm cell strainer (BD Biosciences, New Jersey, USA). Then, 5×10^6^ cells were seeded into 5 cm culture dishes and initially incubated at 37 °C and 5% CO_2_. After 24 h, cultures were washed with PBS to eliminate non-adherent cells. The attached cells were cultured for 10 to 15 d with a-MEM supplemented with 20% FBS, 2mM L-glutamine, 100 U/ml penicillin and 100 mg/ml streptomycin (Invitrogen, Carlsbad, CA, USA). Passage 1 and 2 were used in all experiments.

### CFU assay

To assess the CFU efficiency of BMMSCs, 1×10^3^ primary cultured BMMSCs derived from each experimental group of mice (n=5) were seeded in 5 cm culture dishes (Corning, Lowell, MA, USA) and cultured in proliferation or osteogenic differentiation medium for 14 days. Then, the newly formed colonies were visualized with 0.1% toluidine blue or Alizarin Red/ALP staining following 4% paraformaldehyde fixation for CFU-F and CFU-Ob assay. Aggregates of 50 or more cells were scored as colonies under the microscope (Leica Microsystems, Heerbrugg, Switzerland). CFU efficiency was determined by the number of colonies relative to the total number of seeded cells in each plate. Experiments were performed in triplicate.

### β-galactosidase staining assays

To assess the senescence of the cultured BMMSCs after 48 h (both 4 mon and 16 mon), β-galactosidase staining was determined via a staining Kit (Beyotime Institute of Biotechnology, Jiangsu China) according to the manufacturer's protocol. Experiments were performed in triplicate.

### Real-time PCR analysis

Total RNA was isolated from cultured BMMSCs and from femur bone tissues using Trizol (Invitrogen) according to the manufacturer's instructions. MiRNA was extracted with the mirVana miRNA Isolation Kit (Ambion, Austin, *TX*, USA). The conversion of miRNA and mRNA into cDNA and the detection of miRNAs were carried out according to the manufacturer's instructions using the miScript Reverse Transcription Kit and the miScript SYBR Green PCR Kit (Takara Bio Inc., Shiga, Japan), respectively. Sequences were determined with the CFX96 Real-Time System (Bio-Rad, CA, USA). The optimized miRNA-specific primers for has-miR-17 and the endogenous control U6 were commercially obtained (RiboBio Co., Guangzhou, China). Primary-miR-17 and GAPDH as endogenous control were commercially purchased (Invitrogen). The expression levels of p16, p21, p53, Runx2, ALP, Osterix, TCF3 and Smurf1 (Takara Bio Inc.) were examined. The primers were listed in Table 1. Experiments were performed in triplicate.

**Table 1:** Primers for Real-time PCR

Primer name	Sequence (5′ to 3′)	Temperature (°)	Length (bp)
U6	F:5′GCTTCGGCAGCACATATACTAAAAT3′ R:5′CGCTTCACGAATTTGCGTGTCAT3′	60	89
β-actin	F:5′ TGGCACCCAGCACAATGAA3′ R:5′ CTAAGTCATAGTCCGCCTAGAAGCA 3′	62	186
p53	F:5′ GCTTTGAGGTGCGTGTTTGTG3′ R:5′ TTGGGCAGTGCTCGCTTAG 3′	60	126
p16	F:5′ GCTTCCTGGACACGCTGGT 3′ R:5′ CATCTATGCGGGCATGGTTA3′	60	174
p21	F:5′ GGGAGCAGGCTGAAGGGT3′ R:5′ CGGCGTTTGGAGTGGTAGAA 3′	60	97
Runx2	F:5′ CACTGGCGCTGCAACAAGA 3′ R:5′ CATTCCGGAGCTCAGCAGAATAA 3′	60	127
ALP	F:5′ CCTTGTAGCCAGGCCCATTG3′ R:5′ GGACCATTCCCACGTCTTCAC 3′	60	137
Osterix	F:5′ TGGCGTCCTCCCTGCTTG 3′ R:5′ TGCTTTGCCCAGAGTTGTTG3′	60	125
Smurf1	F:5′ CGTGGGGAAGAAGGTTTGG 3′ R:5′ TGGTCGGGGTTGATTGAAGA 3′	60	158
TCF3	F:5′ AATAACTTCTCGTCCAGCCCTT 3′ R:5′ CTCGTCCAGGTGGTCTTCTATCT 3′	60	159

### Western blot analysis

BMMSCs were harvested in RIPA lysis buffer (Beyotime Institute of Biotechnology) and whole-cell protein extracts were quantified by a BCA assay, separated on SDS-PAGE 8%-12%, and then transferred to PVDF membranes (Millipore, Billerica, MA, USA). Antibodies included p16ink4 (1:800, Abbiotec, San Diego, USA), p21 (1:500, Abcam, Cambridge, England), p53 (1:500, Cell Signaling Technology, Boston, MA, USA), Runx2 (1:500, Abcam), ALP (1:800, Abcam), OSX (1:500, Santa Cruz Biotechnology, Santa Cruz, CA, USA), TCF3 (1:500, Abcam), Smurf1 (1:500, Abcam). In addition, stripped membranes were reprobed with β-actin (1:5000, Abcam) as loading control. Signal detection was performed using the ECL Kit after incubation with an anti-rabbit or anti-mouse IgG secondary antibody (1:5000, CoWin Bioscience Co., Beijing, China). The relative band intensities in the scanned images were analyzed with Image J software (National Institutes of Health, Maryland, USA). Experiments were performed in triplicate.

### Osteogenic differentiation assays

BMMSCs were incubated with osteogenic medium containing 100 nM dexamethasone, 50 mg/ml ascorbic acid and 1 mM b-glycerophosphate for 1 to 2 weeks according to the manufacturer's instructions. To assess osteogenic differentiation, cells were fixed with 60 % isopropanol after 14 d in culture, stained with 1 % alizarin red (Sigma-Aldrich, St. Louis, MO, USA) and lysed in hexadecylpyridinium chloride. Then, the quantification of alizarin red staining intensity was determined with a microplate reader (Bio-TEK Instruments, Winooski, VT, USA) by absorbance at 570 nm. Experiments were performed in triplicate.

### Lentiviral vector construction and transduction

To construct a lentiviral vector for mouse transformation-related protein 53 (p53), p53 was amplified from mouse cDNA via PCR. Primers for lentiviral construct transduction were as follows:

m-tp53-SalI acgGTCGACggATGACTGCCATGGAGGAGTC
m-tp53-NotI ATAAGAATGCGGCCGCcagTCAGTCTGAGTCAGG

The PCR product was digested with Sal I and Not I and inserted into the pLenti 6.3/v5-DEST vector (Invitrogen). The inserted fragments were verified by Sanger sequencing. A lentiviral construct containing the scrambled p53 sequence was used as negative control. The lentivirus was produced by co-transfecting 293T cells with the transfer vector and two packaging vectors (psPAX2, pMD2.G). The virus was subsequently purified by ultracentrifugation. 1×10^5^ BMMSCs were plated in 6-well plates and transduced with lentiviral constructs and 5 μg/ml polybrene (Sigma). Experiments were performed in triplicate.

### Transfection assay

MiR-17 mimics and inhibitor (Ribobio, Guangdong, China) were transfected into BMMSCs at a concentration of 50 nM with the siPORT NeoFX Transfection Agent (Ambion). The medium was replaced 8 h later and the cells were harvested for mRNA analysis after 24 h of transfection and for protein analysis after 48 h of transfection.

SiRNA duplex oligonucleotides against mouse Smurf1 and the negative control (Gene-Pharma Co., Shanghai, China) were chemically modified (2′-O-Methyl) and transfected into the cells at a final concentration of 100 nM using the siPORT NeoFX (Ambion). Experiments were performed in triplicate.

### ELISA assay

TNF-α and IFN-γ serum levels were measured by ELISA using murine TNF-α and IFN-γ assay kits (Neobioscience technology, Shenzhen, China). Experiments were performed in triplicate according to the manufacturer's protocol.

### In vivo bone formation assay

For a single transplant complex, BMMSCs were transduced with a pLenti-p53 lentiviral construct as described above and then cultured for 3 d. 2 × 10^6^ cells were mixed with 15 mg HA/TCP ceramic particles (Sigma-Aldrich) and implanted into subcutaneous pockets on the backs of the 8-weeks NOD/SCID mice (Fourth Military Medical University). As control, BMMSCs from the same sources treated with the lentivirus control were implanted into the other side of the same host's back. The implants were taken out 8 weeks after transplantation, fixed with 4% paraformaldehyde and decalcified with buffered 10% EDTA (pH 6.0). For histological analyses, the sections were stained with HE or Masson's Trichrome (BaSO Diagnostic Inc, Guangdong, China) according to the manufacturer's instructions. Experiments were performed in triplicate.

### Statistics

Statistical evaluation was performed using a t-test and one-way ANOVA for experiments comprising more than three groups, respectively. The data are presented as mean ± SD. *P*< 0.05 was considered statistically significant. The *p*-values were adjusted using the Bonferroni method. Each experiment was repeated three times. Analytic tests were performed using SPSS17.0 Software.

## SUPPLEMENTARY METHODS, FIGURES



## Abbreviations

ALP: Alkaline phosphatase
BCA: Bicinchoninic acid
BMMSCs: Bone marrow mesenchymal stem cells
BMD: Bone mineral density
BMP: Bone morphogenetic protein
CFU-F/-Ob: Colony forming unit-fibroblast/-osteoblast
EDTA: Ethylenediaminetetraacetic acid
FBS: Fetal bovine serum
GAPDH: Glyceraldehyde-3-phosphate dehydrogenase
HE: Hematoxylin and eosin
HA/TCP: Hydroxyapatite/tricalcium phosphate
IFN-γ: Interferon gamma
MSC: Mesenchymal stem cell
miRNA: MicroRNA
α-MEM: Minimum Essential Medium α
PDLSC: Periodontal ligament stem cell
PBS: Phosphate buffered saline
PVDF: Polyvinylidene difluoride
Runx2: Runt-related transcription factor 2
Smurf1: Smad ubiquitin regulatory factor one
siRNA: Small interfering RNA
BV/TV: Trabecular bone volume fraction relative to tissue volume
TCF3: Transcription factor 3
MiRNA: microRNA
MTT: 3-(4,5-dimethylthiazol-2yl)-2,5-diphenyltetrazolium bromide
TNF-α: Tumor necrosis factor alpha

## References

[1] Anton B, Vitetta L, Cortizo F, Sali A (2005). Can we delay aging? The biology and science of aging. Ann N Y Acad Sci.

[2] Wilson A, Shehadeh LA, Yu H, Webster KA (2010). Age-related molecular genetic changes of murine bone marrow mesenchymal stem cells. BMC Genomics.

[3] Cheng H, Qiu L, Ma J, Zhang H, Cheng M, Li W, Zhao X, Liu KY (2011). Replicative senescence of human bone marrow and umbilical cord derived mesenchymal stem cells and their differentiation to adipocytes and osteoblasts. Mol Biol Rep.

[4] Despars G, Carbonneau CL, Bardeau P, Coutu DL, Beauséjour CM (2013). Loss of the osteogenic differentiation potential during senescence is limited to bone progenitor cells and is dependent on p53. PLoS One.

[5] Picinich SC, Mishra PJ, Mishra PJ, Glod J, Banerjee D (2007). The therapeutic potential of mesenchymal stem cells. Cell- & tissue-based therapy. Expert Opin Biol Ther.

[6] Michaloglou C, Vredeveld LC, Soengas MS, Denoyelle C, Kuilman T, van der Horst CM, Majoor DM, Shay JW, Mooi WJ, Peeper DS (2005). BRAFE600-associated senescence-like cell cycle arrest of human naevi. Nature.

[7] Collado M, Serrano M (2010). Senescence in tumours: evidence from mice and humans. Nat Rev Cancer.

[8] Tchkonia T, Zhu Y, van Deursen J, Campisi J, Kirkland JL (2013). Cellular senescence and the senescent secretory phenotype: therapeutic opportunities. J Clin Invest.

[9] Park JS, Kim HY, Kim HW, Chae GN, Oh HT, Park JY, Shim H, Seo M, Shin EY, Kim EG, Park SC, Kwak SJ (2005). Increased caveolin-1, a cause for the declined adipogenic potential of senescent human mesenchymal stem cells. Mech Ageing Dev.

[10] Terai M, Uyama T, Sugiki T, Li XK, Umezawa A, Kiyono T (2005). Immortalization of human fetal cells: the life span of umbilical cord blood-derived cells can be prolonged without manipulating p16INK4a/RB braking pathway. Mol Biol Cell.

[11] Moerman EJ, Teng K, Lipschitz DA, Lecka-Czernik B (2004). Aging activates adipogenic and suppresses osteogenic programs in mesenchymal marrow stroma/stem cells: the role of PPAR-gamma2 transcription factor and TGF-beta/BMP signaling pathways. Aging Cell.

[12] Wall ME, Bernacki SH, Loboa EG (2007). Effects of serial passaging on the adipogenic and osteogenic differentiation potential of adipose-derived human mesenchymal stem cells. Tissue Eng.

[13] Tokalov SV, Grüner S, Schindler S, Wolf G, Baumann M, Abolmaali N (2007). Age-related changes in the frequency of mesenchymal stem cells in the bone marrow of rats. Stem Cells Dev.

[14] Zhou S, Greenberger JS, Epperly MW, Goff JP, Adler C, Leboff MS, Glowacki J (2008). Age-related intrinsic changes in human bone-marrow-derived mesenchymal stem cells and their differentiation to osteoblasts. Aging Cell.

[15] Pan Z, Yang J, Guo C, Shi D, Shen D, Zheng Q, Chen R, Xu Y, Xi Y, Wang J (2008). Effects of hindlimb unloading on ex vivo growth and osteogenic/adipogenic potentials of bone marrow-derived mesenchymal stem cells in rats. Stem Cells Dev.

[16] Tyner SD, Venkatachalam S, Choi J, Jones S, Ghebranious N, Igelmann H, Lu X, Soron G, Cooper B, Brayton C, Park SH, Thompson T, Karsenty G (2002). p53 mutant mice that display early ageing-associated phenotypes. Nature.

[17] Vousden KH, Lu X (2002). Live or let die: the cell's response to p53. Nat Rev Cancer.

[18] Levine AJ (1997). p53, the cellular gatekeeper for growth and division. Cell.

[19] Sharpless NE, DePinho RA (2002). p53: good cop/bad cop. Cell.

[20] Bartel DP (2004). MicroRNAs: genomics, biogenesis, mechanism, and function. Cell.

[21] Croce CM, Calin GA (2005). miRNAs, cancer, and stem cell division. Cell.

[22] Cheng AM, Byrom MW, Shelton J, Ford LP (2005). Antisense inhibition of human miRNAs and indications for an involvement of miRNA in cell growth and apoptosis. Nucleic Acids Res.

[23] Huang J, Zhao L, Xing L, Chen D (2010). MicroRNA-204 regulates Runx2 protein expression and mesenchymal progenitor cell differentiation. Stem Cells.

[24] Zhang JF, Fu WM, He ML, Xie WD, Lv Q, Wan G, Li G, Wang H, Lu G, Hu X, Jiang S, Li JN, Lin MC (2011). MiRNA-20a promotes osteogenic differentiation of human mesenchymal stem cells by co-regulating BMP signaling. RNA Biol.

[25] Liu Y, Liu W, Hu C, Xue Z, Wang G, Ding B, Luo H, Tang L, Kong X, Chen X, Liu N, Ding Y, Jin Y (2011). MiR-17 modulates osteogenic differentiation through a coherent feed-forward loop in mesenchymal stem cells isolated from periodontal ligaments of patients with periodontitis. Stem Cells.

[26] Liu W, Liu Y, Guo T, Hu C, Luo H, Zhang L, Shi S, Cai T, Ding Y, Jin Y (2013). TCF3, a novel positive regulator of osteogenesis, plays a crucial role in miR-17 modulating the diverse effect of canonical Wnt signaling in different microenvironments. Cell Death Dis.

[27] Yan HL, Xue G, Mei Q, Wang YZ, Ding FX, Liu MF, Lu MH, Tang Y, Yu HY, Sun SH (2009). Repression of the miR-17-92 cluster by p53 has an important function in hypoxia-induced apoptosis. EMBO J.

[28] Tanzer A, Stadler PF (2004). Molecular evolution of a microRNA cluster. J Mol Biol.

[29] Khosla S, Riggs BL (2005). Pathophysiology of age-related bone loss and osteoporosis. Endocrinol Metab Clin North Am.

[30] Raisz LG (2005). Pathogenesis of osteoporosis: concepts, conflicts, and prospects. J Clin Invest.

[31] Kurowska-Stolarska M, Alivernini S, Ballantine LE, Asquith DL, Millar NL, Gilchrist DS, Reilly J, Ierna M, Fraser AR, Stolarski B, McSharry C, Hueber AJ, Baxter D (2011). MicroRNA-155 as a proinflammatory regulator in clinical and experimental arthritis. Proc Natl Acad Sci U S A.

[32] Tili E, Michaille JJ, Wernicke D, Alder H, Costinean S, Volinia S, Croce CM (2011). Mutator activity induced by microRNA-155 (miR-155) links inflammation and cancer. Proc Natl Acad Sci U S A.

