# Correction for: The p53/miR-17/Smurf1 pathway mediates skeletal deformities in an age-related model via inhibiting the function of mesenchymal stem cells

**DOI:** 10.18632/aging.205986

**Published:** 2024-06-15

**Authors:** Wenjia Liu, Meng Qi, Anna Konermann, Liqiang Zhang, Fang Jin, Yan Jin

**Affiliations:** 1 State Key Laboratory of Military Stomatology, Center for Tissue Engineering, School of Stomatology, The Fourth Military Medical University, Xi’an, Shaanxi 710032, People’s Republic of China; 2 Research and Development Center for Tissue Engineering, Fourth Military Medical University, Xi’an, Shaanxi 710032, People’s Republic of China; 3 Department of Orthodontics, Medical Faculty, University of Bonn, Bonn, Germany; 4 State Key Laboratory of Military Stomatology, Department of Orthodontic, School of Stomatology, The Fourth Military Medical University, Xi’an, Shaanxi 710032, People’s Republic of China

**Keywords:** aging, mesenchymal stem cells, osteogenesis, p53, miR-17

**Abbreviations:** ALP: Alkaline phosphatase; BCA: Bicinchoninic acid; BMMSCs: Bone marrow mesenchymal stem cells; BMD: Bone mineral density; BMP: Bone morphogenetic protein; CFU-F/-Ob: Colony forming unit-fibroblast/-osteoblast; EDTA: Ethylenediaminetetraacetic acid; FBS: Fetal bovine serum; GAPDH: Glyceraldehyde-3-phosphate dehydrogenase; HE: Hematoxylin and eosin; HA/TCP: Hydroxyapatite/tricalcium phosphate; IFN-γ: Interferon gamma; MSC: Mesenchymal stem cell; miRNA: MicroRNA; α-MEM: Minimum Essential Medium α; PDLSC: Periodontal ligament stem cell; PBS: Phosphate buffered saline; PVDF: Polyvinylidene difluoride; Runx2: Runt-related transcription factor 2; Smurf1: Smad ubiquitin regulatory factor one; siRNA: Small interfering RNA; BV/TV: Trabecular bone volume fraction relative to tissue volume; TCF3: Transcription factor 3; MiRNA: microRNA; MTT: 3-(4,5-dimethylthiazol-2yl)-2,5-diphenyltetrazolium bromide; TNF-α: Tumor necrosis factor alpha.

**This article has been corrected:** The authors recently found that they used a non-representative image of Alizarin red staining in **Figure 6E**, “Alizarin red staining after osteogenic induction for 14 d of BMMSCs derived from old mice with/without siRNA-downregulated Smurf1 level and with/without transfection with miR-17 inhibitor.” They replaced panel 6E with the correct image of Alizarin red staining from the original experiments and state that this correction has no impact on the experimental outcome or conclusions. The authors would like to apologize for any inconvenience caused.

The authors also acknowledged that the email address of the corresponding author, Fang Jin, has been updated to jinfang@fmmu.edu.cn.

The corrected version of **Figures 6** is provided below.

**Figure 6:**
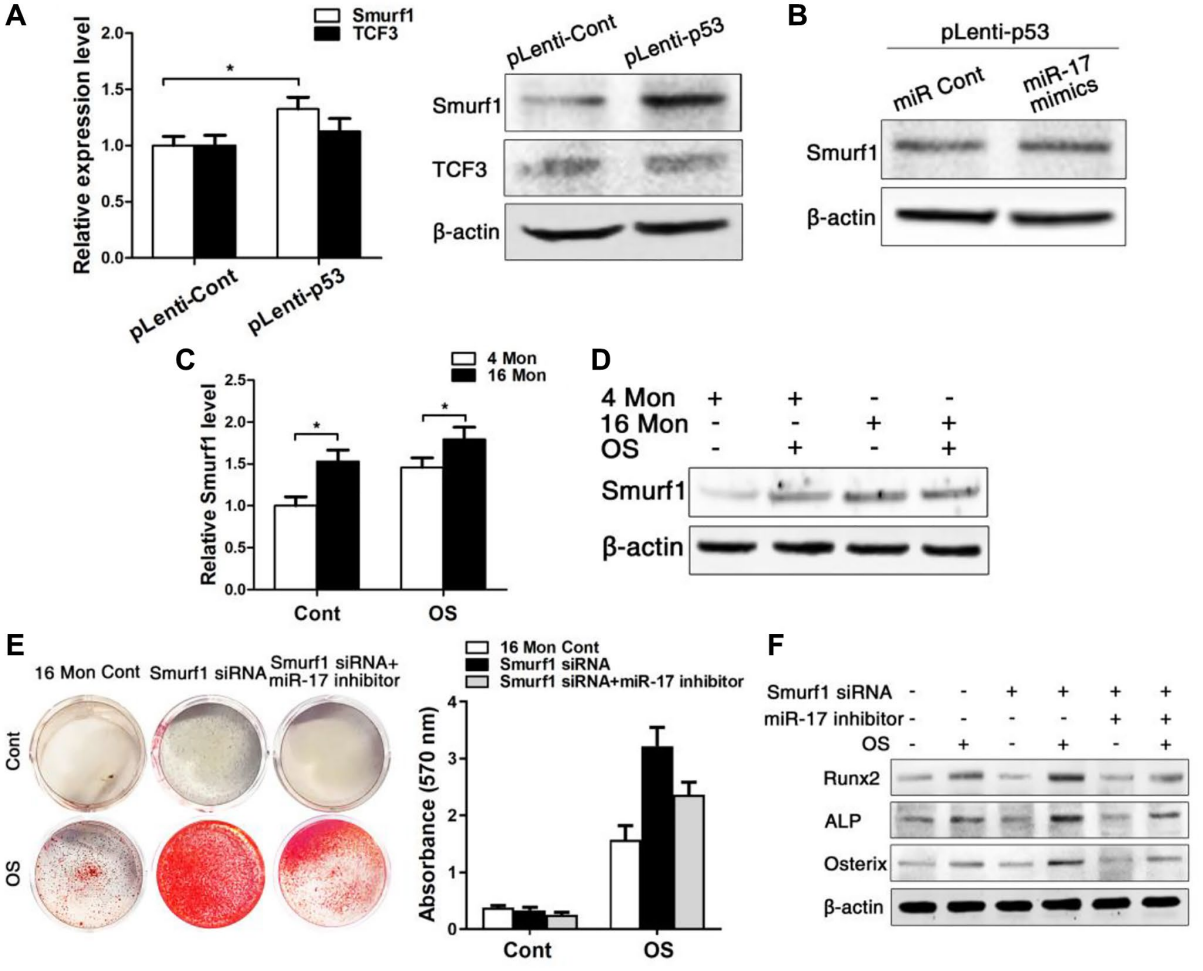
**Smurf1 plays an important role in miR-17- mediated osteogenic differentiation of BMMSCs.** BMMSCs were lentivirally transduced to upregulate the expression level of P53 (= pLenti-P53) or were transduced as lentiviral control (= pLenti-Cont). miR-17 was stable upregulated in BMMSCs by miR-17 mimics (= miR-17 mimics). miRNA control (= miR Cont). 16 Mon Cont = control BMMSCs, Smurf1 siRNA = downregulated Smurf1 level via si-RNA, miR-17 inhibitor = transfection with anti-miR-17. Statistically analyzed values show the mean ± SD (*n* = 10). ^*^p < 0.05. (**A**) Real-time PCR and western blot analysis on the expression of Smurf1 and TCF3 after upregulation of p53 (pLenti-p53) in BMMSCs derived from young mice. Normalization to β-actin. (**B**) Western blot analysis on the expression of Smurf1. Transfection of miR-17 mimics in stable upregulated p53 BMMSCs derived from young mice. Normalization to β- actin. (**C**, **D**) Real-time PCR and western blot analysis of Smurf1 expression in osteogenically differentiated BMMSCs from young and old mice. Normalization to β- actin. (**E**) Alizarin red staining after osteogenic induction for 14 d of BMMSCs derived from old mice with/without siRNA-downregulated Smurf1 level and with/without transfection with miR-17 inhibitor. (**F**) Western blot analysis on old BMMSCs with/without siRNA-down- regulated Smurf1 level and with/without transfection with anti-miR-17 for the osteogenic markers Runx2, ALP, osterix. Normalization to β-actin.

