# Supplementary material for: The p53/miR-17/Smurf1 pathway mediates skeletal deformities in an age-related model via inhibiting the function of mesenchymal stem cells
Source: Aging (Albany NY). 2015 Mar 7;7(3):205–16. doi: 10.18632/aging.100728 (PMC4394731; doi:10.18632/aging.100728)
Supplement: Supplementary file 1 [file aging-07-0205-s001.pdf]

SUPPLEMENTARY METHODS

Proliferation assays. For proliferation analyses of BMMSCs, 5x10^3 cells/well were cultured in 96-well plates. A MTT assay was carried out for 8 d according to the manufacturer´s protocol (Sigma). Absorbance was determined at 490 nm with a microplate reader (Bio-TEK Instruments). Furthermore, cell cycle analysis was performed on PDLSCs and BMMSCs after harvesting single cell suspensions of both. Cells were therefore fixed in ice-cold 75% ethanol 4°C for 24-48 h, washed twice with PBS, stained with 100mg/ml propidium iodide at 4°C for 30 min and subjected to cell cycle analysis using an Elite ESP flow cytometer (Beckman Coulter, Fullerton, CA, USA). Experiments were performed in triplicate.

SUPPLEMENTARY FIGURES

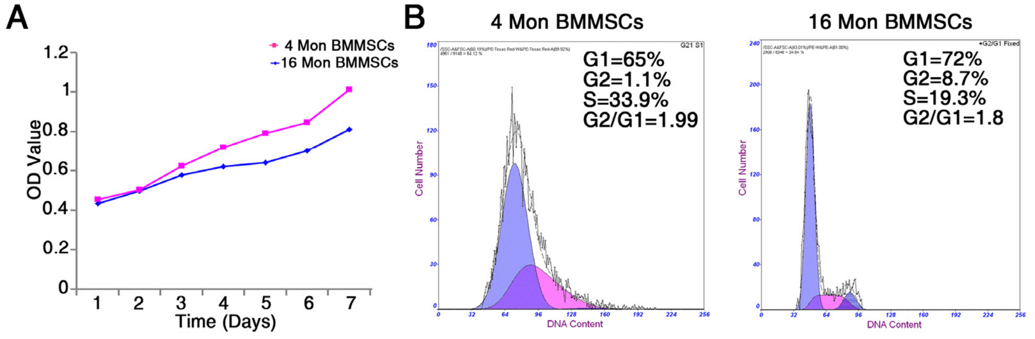

Supplementary Figure 1. Proliferation capacity of BMMSCs from young and old mice. Identification of similar proliferation rates of BMMSCs by means of MTT assay **(A)** and flow cytometric cell cycle analysis (**B**). Exemplary illustration of the data derived from n=3.

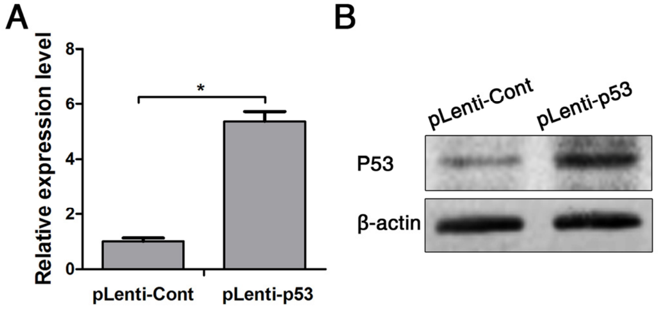

Supplementary Figure 2. p53 transfection efficiency. Real-time PCR **(A)** and western blot **(B)** analyses on p53 expression in BMMSCs from 4 month-old mice after transfection (pLenti-P53). Normalization to ß-actin. Statistically analyzed values show the mean ± SD (n=3). * $p < 0.05$.

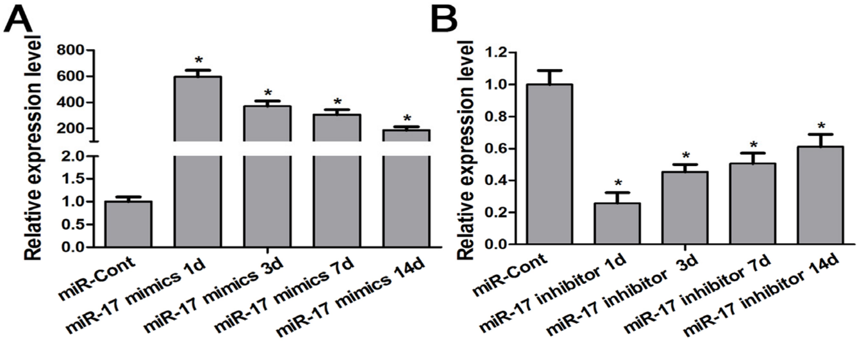

Supplementary Figure. 3 miR-17 mimics and miR-17 inhibitor transfection efficiency. Verification of the microRNA transfection efficiency by transfection of BMMSCs with miR-17 mimics and control, miR-17 inhibitor and control for 1d, 3d, 7d and 14d. Analysis of miR-17 expression levels via real-time PCR at indicated time points. **(A)** Expression level of miR-17 mimics. (**B)** Expression level of miR-17 inhibitor.

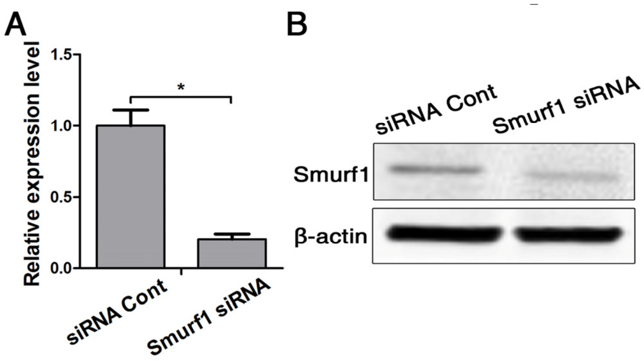

Supplementary Figure 4. Si-Smurf1 transfection efficiency. Real-time PCR **(A)** and western blot **(B)** analyses on Smurf1 expression in BMMSCs from 16 month-old mice after transfection with Smurf1 siRNA. Normalization to ß-actin. Statistically analyzed values show the mean ± SD (n=3). * $p < 0.05$.
